# Psychotic and schizotypal symptoms in non-psychotic patients with obsessive-compulsive disorder

**DOI:** 10.1186/s12888-015-0502-1

**Published:** 2015-05-28

**Authors:** Stian Solem, Kristen Hagen, Christoffer Wenaas, Åshild T. Håland, Gunvor Launes, Patrick A. Vogel, Bjarne Hansen, Joseph A. Himle

**Affiliations:** 1 Department of Psychology, Norwegian University of Science and Technology, 7491 Trondheim, Norway; 2 Divison of Psychiatry, St. Olavs University Hospital, Trondheim, Norway; 3 Clinic of Mental Health, Psychiatry and Addiction Treatment, Sørlandet Hospital HF, Kristiansand, Norway; 4 Department of psychiatry, Haukeland University Hospital, Bergen, Norway; 5 Department of Psychiatry, University of Michigan, Ann Arbor, MI USA; 6 School of Social Work, University of Michigan, Ann Arbor, MI USA

**Keywords:** OCD, Psychosis, Schizotypal, ERP

## Abstract

**Background:**

Research is scarce with regard to the role of psychotic and schizotypal symptoms in treatment of obsessive-compulsive disorder (OCD). The aim of the current study was to investigate the occurrence and specificity of psychotic and schizotypal symptoms among non-psychotic OCD patients, and to examine whether such symptoms was associated with response to exposure and response prevention (ERP), and whether ERP for OCD had an impact on psychotic and schizotypal symptoms.

**Methods:**

Non-psychotic OCD patients (*n* = 133) and a general non-psychotic psychiatric outpatient sample (*n =* 110) were assessed using self-report inventories before and after psychological treatment.

**Results:**

Non-psychotic OCD patients did not report greater degree of psychotic or schizotypal symptoms than the control group. Psychotic and schizotypal symptoms were not associated with OCD symptoms before or after ERP. Psychotic and schizotypal symptom were significantly reduced following ERP.

**Conclusions:**

Psychotic and schizotypal symptoms seem to be equally prevalent among non-psychotic OCD patients and non-psychotic psychiatric controls. These symptoms were more linked to depressive symptoms than OCD symptoms. In non-psychotic OCD patients, ERP seems sufficient in reducing OCD symptoms despite the presence of psychotic- and schizotypal symptoms, and reductions in psychotic- and schizotypal symptoms were observed following ERP.

## Background

Obsessive-compulsive disorder (OCD) and psychotic disorders exhibit both biological and phenomenological overlap [1]. Furthermore, there might not be a clear distinction in OCD between delusions and obsessions as OCD patients describe varying degrees of overvalued ideas [2]. Studies also indicate that obsessions can transform into delusions [3], and that OCD and symptoms of OCD can be associated with the development of psychotic disorder over time [4]. An increased prevalence of OCD in patients with first-episode psychosis has also been found [5]. Although this may seem to link OCD and psychotic symptoms, other studies show mixed evidence concerning this relation, leaving unanswered the question of whether psychotic and schizotypal symptoms are particularly associated with OCD, or whether they constitute symptoms that are just as prevalent in several other emotional disorders.

Some studies have explored psychotic symptoms in patients with OCD [4, 6–10]. It has been argued that a symptom-based approach may be more favourable than a diagnostic approach due to questionable validity of diagnostic systems, substantial variation in symptomatology within categorical diagnosis and the ignoring of likely continua in the symptomatology between normal and pathological [11]. However, there is great variety in the reported occurrence of such symptoms, ranging from zero to fifty percent [4].

There have been proposals of a distinct subtype of schizotypal OCD [3, 6, 12], as OCD patients with schizotypal symptoms have poorer insight and lower functioning than OCD patients without such symptoms. However, there are discrepant findings. One study found that patients with depression, panic disorder, social phobia, post-traumatic stress disorder, or alcohol/substance dependency, had higher life-time prevalence of schizotypal personality disorder than patients with OCD [7]. A second study found that patients with OCD reported more symptoms of schizotypal disorder than patients with depression, but less so than patients with bipolar disorder or schizophrenia [13]. A third study found that OCD patients with schizotypal symptoms reported higher rates of comorbid major depression, post-traumatic stress disorder, substance use disorders, and greater general psychopathology [14]. They did not find any associations between schizotypal symptoms and OCD when controlling for differences in demographics and comorbidity.

To further complicate matters, a longitudinal study argued that sub-clinical levels of psychotic disorders are associated with development of anxiety, emotional and substance-related disorders [15]. Unlike the studies mentioned above, this study indicated that such an association was stronger between OCD and psychotic symptoms, than any of the other disorders. The proposed association between symptoms of OCD and psychotic and schizotypal symptoms may also be moderated by symptom severity [16] because low levels of schizotypal and OCD symptoms were distinct from each other, whereas among high scoring individuals the two symptom categories were associated. Thus, it remains uncertain if psychotic and schizotypal symptoms are linked to OCD.

The presence of psychotic and schizotypal symptoms may influence treatment outcome in OCD. Indeed, previous studies indicate that OCD patients with comorbid schizotypal disorder show poorer response to medical treatment for OCD [3]. Related research comes from studies on poor insight in OCD. OCD with poor insight has been described as egosyntonic symptoms that might extend to delusions and psychosis [17]. Most likely, insight falls on a continuum and is associated with symptom severity, chronicity, and poorer treatment prognosis [18]. There is still a great lack of knowledge with regard to psychotic and schizotypal symptoms among patients with OCD and how these symptoms affect psychological treatment of OCD. In addition, we do not know whether treatment for OCD reduces psychotic and schizotypal symptoms.

In the current study we assessed psychotic and schizotypal symptoms among non-psychotic patients with OCD. Furthermore, this study aimed to elucidate whether such symptoms are elevated in the OCD population, or similarly present in a general non-psychotic psychiatric outpatient sample. Finally, the current study aimed to investigate whether such symptoms were associated with reduced OCD treatment response, and if these symptoms are reduced following exposure and response prevention treatment (ERP).

## Methods

### Participants and procedure

This study included two samples: non-psychotic patients diagnosed with OCD (*n* = 133) and a general non-psychotic psychiatric outpatient group (*n* = 110). Participants with psychotic disorder, suicidal behaviour, and alcohol or substance dependency were excluded. Before excluding OCD patients, there were 182 treatment seekers, but 49 of these were excluded. A total of seven patients were excluded from the OCD group due to psychotic disorders. The number of excluded patients from the control group is unknown. Due to missing data the sample sizes were reduced to 103 in the non-psychotic OCD group and 73 for the non-psychotic control group at post-treatment.

The non-psychotic OCD group was comprised of patients from two previously published treatment studies [19, 20], and an unpublished multi-center treatment study. The mean age was 34.7 years (*SD*: 11.6) and consisted of 71.5 % (n = 82) female participants. Seven patients in the non-psychotic OCD group were excluded due to psychosis. OCD patients were diagnosed using the Structural Clinical Interview for DSM IV Axis I Disorders [21] or Anxiety Disorder Interview Schedule-Revised [22]. Patients received either ERP-based group therapy over a period of 12 weeks [19] or individual ERP based on the manual by Foa [23]. A total of 65 received group treatment and 68 had individual ERP treatment. Independent t-tests found no significant difference between people in group and individual treatment with regard to changes in symptoms of paranoia (*p* = .19), psychoticism (*p* = .36), schizotypal signs (*p* = .17), and schizophrenia nuclear signs (*p* = .17).

The main ingredients of ERP were in the first session to formulate a case-conceptualization, present a habituation rationale, and use of self-registration of rituals for homework. The second session involved creating the exposure hierarchy and introducing the rules for ritual prevention. The following sessions were similar in structure and consisted mainly of checking homework assignments, in vivo and imaginary exposure delivered in a sequence as specified by the hierarchy and agreeing on homework assignments. Focus turned to relapse prevention when treatment was approaching termination.

The general non-psychotic psychiatric outpatient group consisted of patients from a university clinic. They received 15 weekly sessions of eclectic psychotherapy delivered by graduate psychology students under supervision by experienced psychologists [24]. The general outpatient group had individual treatment only. Supervisors had different backgrounds with training in CBT or psychodynamic therapies. Supervisors were to ensure that treatment should be based on good clinical research practice. Choice of treatment was made in agreement between therapists and supervisors. The average age in this sample was 34.5 years (*SD*: 11.4) and 74.5 % (n = 82) were female. Exclusion criteria included psychosis, violent behaviour, serious substance abuse or suicidal behaviour. Seven participants from the control group were excluded from our analyses due to being diagnosed with OCD. Participants in the non-psychotic control group were diagnosed using Mini International Neuropsychiatric Interview (MINI) [25], by graduate students under supervision and observation by experienced clinical psychologists.

The psychiatric control group presented with a variety of different problems:; one with pathological gambling; two with anorexia; three with somatoform disorder; three with hypochondriasis; six with adjustment disorder; one with post-traumatic stress disorder; six with mixed anxiety and depression; nine with generalized anxiety disorder; 11 with panic disorder/agoraphobia; one with specific phobia; 14 with social phobia; 36 with depressive disorders; one with bipolar disorder; one with cannabis related disorders, and 10 people were diagnosed with a personality disorder (in some cases the graduate students did SCID-II interviews in addition to the MINI). The number of patients assessed with SCID-II is unknown. SCID-II was not used as a routine procedure, but administered when the therapist had a hypothesis that the patient suffered from a personality disorder. Twenty participants in the non-psychotic psychiatry control group were not diagnosed with a specific disorder. They were still included in the study as they were treatment seeking and received treatment at the clinic.

For the non-psychotic control group, a one-way ANOVA revealed no significant difference between five different diagnostic groups with regard to scores on psychoticism, *F*(4,102) = 2.113, *p* = .085, or paranoid ideation, *F*(4, 102) = 2.148, *p* = .08. Post-hoc tests using Tukey’s method revealed that the no diagnosis group trended towards lower scores than the depression group on paranoid ideation (*p* = .078) and psychoticism (*p* = .117). The five diagnostic groups consisted of; depressive disorders (n = 28), anxiety disorders (*n* = 39), personality disorders (*n* = 8), several comorbid disorders (*n* = 12), and no diagnosis (*n* = 20).

The non-psychotic OCD group and the non-psychotic psychiatric control group had comparable demographic characteristics. There were no significant difference between the two groups with regard to age, *t*(234) = .22, *p* = .83, and gender, *F*(1, 241) = .29, *p* = .59. The two groups were also comparable with regard to employment status. A considerable proportion of the patients were unemployed or receiving disability benefits as 47.3 % of the psychiatric controls and 48.3 % of the OCD patients were not working or studying. The study was approved by Regional Committee for Medical and Health Research Ethics in Norway. Written consent for participation in the study was obtained from participants.

### Assessments

#### Symptom Checklist 90 Revised (SCL-90-R)

Psychotic- and schizotypal symptoms were measured with the subscales *psychoticism* (example item; “The idea that something is wrong with your mind”) and *paranoid ideation* (example item; “Feeling that you are watched or talked about by others”) of SCL-90-R [26]. The SCL-90-R consists of ninety questions that can be answered on a scale from zero (not at all) to four (extremely). It measures symptoms from the past week. Its factor structure and generalizability has been debated [27], and it has been suggested that the SCL-90-R is a measure of negative affect rather than nine different symptom clusters. Nevertheless, a study has shown that the two subscales psychoticism and paranoid ideation can discriminate patients with psychotic disorder or psychotic symptoms from patients with non-psychotic disorder or psychosis in remission [28]. Furthermore, these subscales, and new subscales consisting of questions from both subscales, have been utilized as measures of both subclinical psychotic and schizotypal symptoms in several studies [15, 29–34].

Two different subscales have been suggested to measure psychotic and schizotypal symptoms using the SCL-90 [33]. The first was labelled *schizotypal signs* (example item; “Feeling others are to blame for most of your troubles”) since it resembled criteria for schizotypal personality disorder, whereas the second was labelled *schizophrenia nuclear symptoms* (example item; “Hearing voices that other people do not hear”) due to its similarities with criteria for schizophrenia. Later studies have validated these two subscales [15, 32] and they are assumed to represent psychotic and schizotypal symptoms in a more adequate and stable manner than the two previously used subscales. They have also been used in meta-studies on psychotic experiences in the normal population [29]. In the current study, both the two original subscales and the two scales suggested by Rössler and colleagues were used.

#### Yale-Brown Obsessive Compulsive Scale (Y-BOCS)

Y-BOCS [35] was utilized to measure symptoms of OCD. Y-BOCS is considered the gold standard for measuring OCD severity. Obsessions and compulsions are rated on a 0–4 scale with regard to frequency, distress, interference, resistance, and control. Only the OCD group was assessed using Y-BOCS.

#### Beck Depression Inventory (BDI)

BDI [36] is one of the most widely used instruments for identification and severity measurement of depressive symptoms. In the current study BDI was employed as a control variable as several authors have argued that depressive symptoms might bias self-rated psychotic symptoms [37]. Only the OCD group was assessed with the BDI.

## Results

### Preliminary analyses

As expected, the non-psychotic OCD group (*M* = 2.02, *SD* = .84) had significantly higher scores than the non-psychotic control group (*M* = 1.32, *SD* = .69) on the OCD scale of the SCL-90, *t*(241) = 6.918, *p* = .000. There were no differences between the two groups on the depression scale of SCL-90 (1.52 [.87] for the non-psychotic OCD group vs. 1.62 [.81] for the non-psychotic psychiatric controls, *t*(241) = −.88, *p* = .38). Also, there were no differences between the samples on the total score of SCL-90 (1.17 [.68] for the non-psychotic OCD group vs. 1.10 [.58] for the non-psychotic psychiatric controls), *t*(241) = .82, *p* = .41.

### Psychotic- and schizotypal symptoms

Independent t-tests comparing the two groups at pre- and post-treatment found no significant differences between them with regard to psychoticism, paranoid ideation signs, schizophrenia nuclear symptoms, or schizotypal signs. As presented in Table 1, the non-psychotic OCD group and the non-psychotic general patient group were very similar both before and after treatment concerning psychotic- and schizotypal symptoms. Lowest scores were obtained on the schizophrenia nuclear symptoms (mean item score of .18 for both groups on a scale of 0–4), while mean item scores of psychoticism was approximately .50 for both groups. Scores on paranoid ideating and schizotypal signs were approximately .80 for both groups.

**Table 1:** Psychotic- and schizotypal symptoms in OCD group (n = 103) and outpatient group (n = 71) before and after treatment

	OCD	GP	Sign.
Psychoticism pre-treatment	.50 (.49)	.54 (.45)	.71
Psychoticism post-treatment	.34 (.39)	.37 (.41)	.96
Paranoid ideation pre-treatment	.85 (.67)	.80 (.69)	.29
Paranoid ideation post-treatment	.56 (.61)	.57 (.61)	.85
Schizophrenia nuclear sxs pre-treatment	.18 (.32)	.18 (.32)	.78
Schizophrenia nuclear sxs post-treatment	.11 (.24)	.12 (.28)	.44
Schizotypal signs pre-treatment	.87 (.70)	.88 (.70)	.40
Schizotypal signs post-treatment	.59 (.61)	.64 (.63)	.80

OCD = OCD group, GP = General psychiatric outpatient sample, sxs = symptoms. Scales range from 0 (not at all) to 4 (extremely)

For the schizophrenia nuclear symptoms, 34.8 % of the OCD group had a confirming score of 1 or more compared to 34.5 % for the control group. This similarity was evident for the other subscales as well; for psychoticism, 88 % of the non-psychotic OCD group had a confirming score compared to 88.2 % in the non-psychotic controls. For paranoid ideation, 90.2 % in the non-psychotic OCD group had a confirming score compared with 85.5 % in the non-psychotic controls. And for schizotypal signs, 94 % in the non-psychotic OCD group had a confirming score compared with 90.9 % of the non-psychotic controls. There was great variation within each scale. Concerning psychotic symptoms, items such as hearing voices that other people do not hear, or the idea that someone else can control your thoughts, only 2.3 and 6.0 % had a score higher than zero. Items such as feeling lonely even when you are with other people and the idea that something is wrong with your mind was more common; as many as 65.2 and 50 % had scores higher than zero. There was somewhat less variation within schizotypal scores. One third reported feeling that others are to blame for most of your troubles, whereas more than two thirds reported having ideas or beliefs that others do not share.

A closer inspection using independent t-tests for the individual subscale items found four significant differences between the two patient groups: having thoughts about sex that bother you a lot (item 84) (*p* = .000), the idea that you should be punished for your sins (item 85, *p* = .006), and having ideas or beliefs that others do not share (item 68, *p* = .000) were more common in the OCD group. The idea that something serious is wrong with your body (item 87, *p* = . 004) was more common in the control group. Put differently, the observed differences between the groups appeared more related to OCD symptoms rather than psychotic symptoms.

### Treatment effects

We conducted repeated measures ANOVA comparing slopes of change for the non-psychotic OCD and control group. For *psychoticism* there was a significant reduction of symptoms, *F*(1,172) = 33.870, *p* = .000, *η*^*2*^ = .165, and no interaction effect *F*(1,172) = .006, *p* = .939. There was not a significant difference in treatment effect between non-psychotic OCD group and controls for psychoticism, *F*(1,172) = .081, *p* = .611, *η*^*2*^ = .002.

For *paranoid ideation* signs there was a significant reduction of symptoms, *F*(1,172) = 36.036, *p* = .000, *η*^*2*^ = .173, and no interaction effect *F*(1,172) = .640, *p* = .425. There was not a significant difference in treatment effect between the non-psychotic OCD group and controls for paranoid ideation signs, *F*(1,172) = .037, *p* = .848, *η*^*2*^ = .000.

For *schizophrenia nuclear symptoms* there was a significant reduction of symptoms, *F*(1,172) = 6.960, *p* = .009, *η*^*2*^ = .039, and no interaction effect *F*(1,172) = .012, *p* = .914. There was not a significant difference in treatment effect between the non-psychotic OCD group and controls for schizophrenia nuclear signs, *F*(1,172) = .032, *p* = .858, *η*^*2*^ = .000.

For *schizotypal signs* there was also a significant reduction of symptoms, *F*(1,170) = 36.727, *p* = .000, *η*^*2*^ = .178, and no interaction effect *F*(1,170) = .265, *p* = .607. Again, there was not a significant difference in treatment effect between the non-psychotic OCD group and controls for schizotypal signs, *F*(1,170) = .111, *p* = .739, *η*^*2*^ = .001.

### Specificity of psychotic and schizotypal symptoms to OCD

Correlation analyses were conducted to further investigate the possible specificity of psychotic and schizotypal symptoms to OCD. Partial correlations were also obtained controlling for the possible confounding effects of depression. The results of this analysis are presented in Table 2. There were weak but significant correlations between OCD symptoms and psychotic and schizotypal symptoms, however, these correlations did not remain significant when controlling for depressive symptoms. A previous study [16] found that schizotypal and OCD symptoms were associated among people reporting severe symptoms. In our study, the correlations were non-significant when only patients with Y-BOCS scores above 28 (*N* = 26) were included. In fact the correlations were negative and ranged from -.14 (*psychoticism, p* = .48) to -.36 (*schizophrenia nuclear symptoms*, *p* = .07).

**Table 2:** Correlations between symptoms of OCD, psychotic symptoms, and depression in the OCD group

		Y-BOCS pre		Y-BOCS post		BDI pre		BDI post	
	α	Bivariate	Partial	Bivariate	Partial	Bivariate	Partial	Bivariate	Partial
Psychotisism pre-treatment	.77	.25**	.05	.17	-.14	.55**	.50**	.58**	.51**
Psychotisism post-treatment	.70	.21*		.32**	.16	.40**	.36**	.61**	.56**
Paranoid ideation pre-treatment	.75	.23*	.05	.16	-.16	.52**	.48**	.54**	.50**
Paranoid ideation post-treatment	.75	.20*		.34**	.19	.35**	.31**	.59**	.55**
Schizotypal signs pre-treatment	.83	.26**	.06	.19*	-.15	.57**	.52**	.58**	.53**
Schizotypal signs post-treatment	.80	.22*		.35**	.19	.41**	.37**	.67**	.63**
Sch. nuclear sxs pre-treatment	.41	.02	.04	.04	.08	.23**	.23**	.19*	.11
Sch. nuclear sxs post-treatment	.04	.08		.16	.11	.13	.11	.20*	.16
BDI pre	.92	.41**		.14				.60**	
BDI post		.14		.40**					

Pre = pre-treatment, Post = post-treatment, BDI = Beck Depression Inventory, Y-BOCS = Yale-Brown Obsessive Compulsive Scale, Sch. Nuclear sxs = schizophrenia nuclear symptoms. Partial correlations control for BDI/Y-BOCS. *p < .05, **p < .01

A final aim of the study was to explore if psychotic and schizotypal symptoms affected treatment outcome for OCD. As evident from Table 2, after controlling for depressive symptoms, there was no association between psychotic- or schizotypal symptoms before treatment and OCD symptoms after treatment. Thus, higher levels of these symptoms did not attenuate the effect of ERP.

## Discussion

Comparison between OCD patients and the general patient sample indicated that there was no significant difference in self-reported psychotic- or schizotypal symptoms. Compared with previous research using healthy controls [27], the non-psychotic OCD group report significantly more psychotic- and schizotypal signs (equal to an effect size of .92 for both scales). The fact that the non-psychotic OCD group and the general patient group resemble each other indicates that such symptoms constitute a general tendency for all psychiatric morbidity and not specifically for OCD. However, this study did not include psychotic patients, and it is therefore possible that OCD patients more often display psychotic comorbidity than other patient groups, but that the exclusion of such patients in the current study did not reveal such a tendency. It is, nevertheless, interesting that among non-psychotic OCD-patients, psychotic- and schizotypal symptoms were related to depressive symptoms, and not OCD symptoms. Scores on psychotic and schizotypal symptoms were moderately correlated with depressive symptoms. There may be considerable overlap between depressive symptoms and some of the SCL-90-R items assessing psychotic and schizotypal symptoms. This corresponds with previous research which has challenged the factor structure of the SCL-90-R. Vassend and Skrondal [27] found psychotic and schizotypal symptoms to be significantly associated with the other symptom dimensions and it was suggested that SCL-90-R may measure general negative affectivity rather than specific symptom dimensions [27]. A previous study [4] found an association between OCD symptoms and psychotic symptoms after controlling for several variables, including depression. Their study did however, not exclude psychotic patients and had a smaller sample of subjects diagnosed with OCD. The lack of such an association in the present study may stem from the fact that OCD symptoms and schizotypal symptoms associate at only high levels of both symptoms [16], and that the overall level of psychotic- and schizotypal symptoms were too low. However we did not find evidence of such when investigating the patients with the most severe OCD symptoms.

In the non-psychotic OCD group, only 6 % reported thought control (the idea that someone else can control your thoughts) and 2 % hearing voices. Other psychotic and schizotypal symptoms were much more prevalent. Such great variation should be expected considering the heterogeneity of psychotic symptoms. In a study by Adam et al. [9] nearly 40 % of 30 OCD patients reported hallucinations or delusions. Differences between these findings and the present study may be due to the exclusion of patients diagnosed with comorbid psychotic disorders. Additionally, the current study assessed symptoms over the past week, whereas the Adam et al. study [9] assessed symptoms in the past twelve months.

The presence of psychotic- and schizotypal symptoms was not associated with reduced response to OCD treatment. Previous studies have found that OCD patients with comorbid schizotypal disorder do not respond as well to ERP [3]. The results from our study, however, suggest that non-psychotic OCD patients with sub-clinical levels of schizotypal disorder or psychotic disorder do not require additional therapeutic interventions beyond standard ERP. However, it was observed that patients with the highest load of schizotypal symptoms had significantly greater depressive symptomatology post-treatment. This may indicate that additional therapeutic intervention for depressive symptoms may be warranted for this subgroup of OCD patients.

A significant reduction in both psychotic- and schizotypal symptoms was observed following treatment and the reductions were similar for the non-psychotic OCD group and the control group. This indicates that ERP also has therapeutic efficacy for psychotic and schizotypal symptoms. Such improvement may be of great importance considering the finding that psychotic symptoms in OCD may increase risk for developing psychotic disorder [4].

The current study has different strengths and limitations. A relatively large size of patients has been examined and contrary to several previous studies, we controlled for depressive symptoms. Limitations include the use of SCL-90-R as the only measure of psychotic or schizotypal symptoms. The validity of the SCL-90-R in assessing schizotypal symptoms in OCD is unclear. Even trained assessors may experience trouble with classifying particular complaints as a symptom of OCD or psychosis. Thus, it seems likely that patients might have difficulties with responding to self-report questionnaires assessing such symptoms. Future research should explore in greater depth the reliability of self-reported psychotic symptoms; although some studies have provided preliminary evidence of reliability and validity [28]. Future research should also explore the relationship between poor insight and psychotic symptoms in OCD. Another limitation is the use of a general psychiatric outpatient sample instead of sub-samples of different specific diagnostic groups. There is a possibility that psychotic and schizotypal symptoms differ across disorders. We did not find any evidence of such differences within our non-psychotic control group, but that could be due to small sample sizes. A final limitation concerns the different methods for determining diagnoses across the samples.

## Conclusions

The presence of self-reported psychotic- and schizotypal symptoms are similar among non-psychotic OCD patients and general non-psychotic psychiatric outpatients. Such symptoms were associated with depressive symptoms rather than OCD symptoms. Sub-diagnostic levels of such symptoms are not associated with reduced treatment response to ERP, in fact ERP is associated with a reduction in both psychotic- and schizotypal symptoms. The reductions in these symptoms were similar for ERP for OCD and for eclectic treatment for psychiatric outpatients.
